# An Audit of the Mental Health Admissions to the Paediatric Assessment Unit (PAU) in the Royal Aberdeen Childrens Hospital (RACH)

**DOI:** 10.1192/bjo.2025.10679

**Published:** 2025-06-20

**Authors:** Billie-Jo Stephen

**Affiliations:** NHS Grampian, Aberdeen, United Kingdom

## Abstract

**Aims:** To determine the number of mental health admissions to the Paediatric Assessment Unit in the Royal Aberdeen Childrens Hospital to provide a basis for implementing improvements to our knowledge and staffing so that we can provide a higher quality service for this group of patients.

**Methods:** Data was collected from 1 June 2023 to 31 May 2024. This period was used as any patient notes prior to 1 June 2023 were documented on paper, thus improving accuracy and consistency in collection method. Mental health admissions also included place of safety. Each patient who was admitted to PAU under these presenting complaints was noted from the nursing staff’s admissions book and the relevant information was then collected from medical notes on the online system 'Trakcare'.

**Results:** There were 4898 admissions to PAU from 1 June 2023 to 31 May 2024. 98 of these admissions were due to mental health. This was 2% of the total admissions to PAU. 38% of patients had a pre-existing mental health diagnosis. The most common presentation was mixed overdose with paracetamol-only overdose being the second most common. The average time spent in PAU was 2.1 days which ranged from 1 day to 20 days. 87% of patients received a psychiatry review from either CAMHS or liaison psychiatry. Of the 98 patients who were admitted, 27% required treatment for paracetamol overdose and 7% required treatment and/or monitoring for anorexia. The remaining patients either received observation only with or without monitoring of vital signs. No patients required emergency psychiatric medications, such as antipsychotics or sedation. 75% of patients were followed up by CAMHS, 3% were admitted and 22% received no follow-up.

**Conclusion:** Mental health accounts for 2% of PAU admissions and can have prolonged stays – up to 20 days found in the audited year. In line with the RCPCH, paediatricians are to have a role in prevention, early recognition and holistic care of mental illness to help with the growing demand in young adults and children. This audit highlights the growing demand of mental health and why health professionals within paediatrics need to develop the experience and confidence to help manage these patients.

